# Biofilms possibly harbor occult SARS-CoV-2 may explain lung cavity, re-positive and long-term positive results

**DOI:** 10.3389/fcimb.2022.971933

**Published:** 2022-09-28

**Authors:** Daqian He, Chaojiang Fu, Mingjie Ning, Xianglin Hu, Shanshan Li, Ying Chen

**Affiliations:** ^1^ Department of Thoracic Surgery I, The Third Affiliated Hospital of Kunming Medical University (Yunnan Cancer Hospital, Yunnan Cancer Center), Kunming, China; ^2^ Emergency Department (Outpatient Chemotherapy Center), The Third Affiliated Hospital of Kunming Medical University (Yunnan Cancer Hospital, Yunnan Cancer Center), Kunming, China; ^3^ Department of Anesthesiology, The Third Affiliated Hospital of Kunming Medical University (Yunnan Cancer Hospital, Yunnan Cancer Center), Kunming, China

**Keywords:** SARS-CoV-2, biofilm, COVID-19, lung cavity, re-positive, co-infection

## Abstract

During the COVID-19 pandemic, there have been an increasing number of COVID-19 patients with cavitary or cystic lung lesions, re-positive or long-term positive nucleic acid tests, but the mechanism is still unclear. Lung cavities may appear at long time interval from initial onset of coronavirus infection, generally during the absorption phase of the disease. The main histopathological characteristic is diffuse alveolar damage and may have more severe symptoms after initial recovery from COVID-19 and an increased mortality rate. There are many possible etiologies of pulmonary cavities in COVID-19 patients and we hypothesize that occult SARS-CoV-2, in the form of biofilm, is harbored in the airway lacuna with other pathogenic microorganisms, which may be the cause of pulmonary cavities and repeated and long-term positive nucleic acid tests.

## Introduction

Novel coronavirus disease 2019 (COVID-19) is a kind of pneumonia caused by severe acute respiratory syndrome coronavirus 2 (SARS-CoV-2) infection. The typical imaging findings of COVID-19 are bilateral grand glass opacities in the lungs (Adams et al., 2020; Chen et al., 2020a; Guan et al., 2020). Cavitary or cystic lung lesions are seldom found and can be ignored from time to time, however, they may lead to serious consequences like pneumothorax (Zoumot et al., 2021; Kalenchic et al., 2022), thus require more attention. Cavitary or cystic lung lesions in COVID-19 patients may be the result of direct SARS-CoV-2 infection, co-infection with bacterial, fungal or mycobacterial pathogens, secondary to co-existing interstitial lung lesions, cystic bronchiectasis, or barotrauma related to mechanical ventilation, malignancy or metastasis (Aggarwal et al., 2021). COVID-19 patient complicated with pulmonary cavities has a wide age span, including infant, child, adult and elderly patient (Chen et al., 2021; Egoryan et al., 2021; Ozgur and Dogan, 2021; Zoumot et al., 2021; He et al., 2022), can appear in both severe and mild COVID-19 (Selvaraj and Dapaah-Afriyie, 2020; Chen et al., 2020a; Afrazi et al., 2021; Aggarwal et al., 2021; Chen et al., 2021; Jafari et al., 2021; Zoumot et al., 2021; Kalenchic et al., 2022). Some scholars consider lung cavity formation as a late complication during COVID-19 recovery (Zoumot et al., 2021; Egoryan et al., 2021). However, whether pulmonary cavity is related to the presence of SARS-CoV-2 and the mechanism of occurrence still remain unclear. Besides, the number of patients with long-term positive nucleic acid tests or re-positive results during COVID-19 convalescence period has soared (He et al., 2020; Lu et al., 2020a; Wang et al., 2020b; Qiao et al., 2020; Lu et al., 2020a; Liang et al., 2021; Wu et al., 2021; Zhu et al., 2021; He et al., 2022; Maccio et al., 2022). The source of SARS-CoV-2 and the mechanism of re-positive have not been clarified either. Herein, we propose a relevant hypothesis based on existing reports.

**Figure 1:**
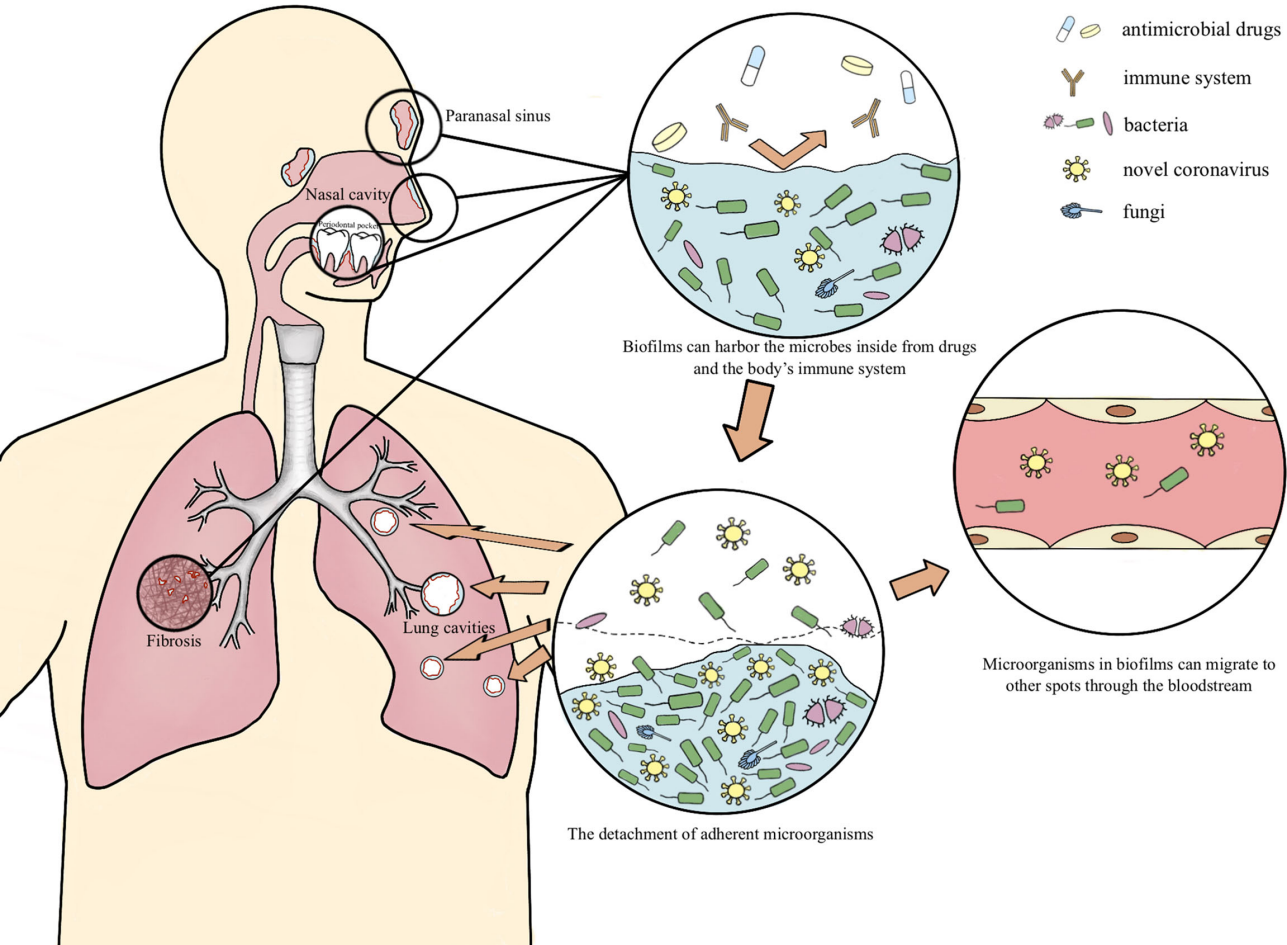
The possible mechanism of the long-term retention of SARS-CoV-2 Occult SARS-CoV-2 may coexist with other pathogenic microorganisms in respiratory lacuna (paranasal sinuses, periodontal clearance, bronchioloalveolar necrotic tissue and pulmonary fibrosis tissue, etc.) in the form of biofilm, and transfers into the lung then multiply when the body is in an immunocompromised situation, as well as migrates to other organs at the distance of the entrance through the bloodstream, accounting for the cavitation, re-positive and long-term positive nucleic acid tests during the COVID-19 rehabilitation.

As an aggregation form of microbes, biofilms are closely associated with nosocomial infections and over 80% bacterial infection is contained to biofilms (Liu et al., 2012; Gomez-Carretero et al., 2017; Sun et al., 2017; Arciola et al., 2018; Strom et al., 2020). According to research, the resistance of biofilm is approximately 100 to 1000 times that of its corresponding planktonic mode, thus obtaining a favorable adaption to adverse external environment. Biofilms can act as a barrier, shielding pathogenic microorganisms from drugs and patients’ immune response (Samrot et al., 2021; Ciofu et al., 2022). Multispecies biofilms exist both externally and within the host in nature (Von Borowski and Trentin, 2021). Fahrenfeld et al. detected the N1 and N2 genes of SARS-CoV-2 in sewage biofilms, which can be considered as a vitro model (Fahrenfeld et al., 2022). Actually, biofilms with viruses have been reported to exist in multiple microbiotas. Multispecies biofilms which encompass Gram-negative bacteria and filamentous fungi and enteric viruses have been spotted, as well as *Candida albicans* biofilms comprised with the coxsackievirus type B5 (CVB5) or herpes simplex virus 1 (HSV-1). Gomes’s team confirmed the presence of SARS-CoV-2 in oral biofilm samples (Gomes et al., 2021; Von Borowski and Trentin, 2021). In this paper, we hypothesize the possible mechanism of the long-term retention of SARS-CoV-2 in the form of biofilm which leads to lung cavities, re-positive and long-term positive nucleic acid tests (**Figure 1**).

## The hypothesis

Occult SARS-CoV-2 may coexist with other pathogenic microorganisms in respiratory lacuna (nasal sinuses, periodontal clearance, bronchioloalveolar necrotic tissue and pulmonary fibrosis tissue, etc.) in the form of biofilm, and transfers into the lung then multiply when the body is in an immunocompromised situation, accounting for the cavitation, re-positive and long-term positive nucleic acid tests during the COVID-19 rehabilitation (**Figure 1**).

### Evaluation of the hypothesis

Abundant cases of SARS-CoV-2 co-infection with other microorganisms have been reported, which were associated with venerable age, immunosuppression, cardiovascular disease, diabetes, intensive care unit attending, mechanical ventilation treatment, long-term antibiotic use, glucocorticoid therapy, prolonged hospitalization, exacerbation of symptoms, and poor prognosis (Bao et al., 2020; Blasco et al., 2020; Contou et al., 2020; Wang et al., 2020a; Alhumaid et al., 2021; He et al., 2021; Yasmin et al., 2021; Gomes et al., 2022; Hedberg et al., 2022; Ortega-Pena et al., 2022; Rouze et al., 2022; SeyedAlinaghi et al., 2022; Shetty et al., 2022). *M. pneumoniae*, *P. aeruginosa*, *H. influenzae*, and *K. pneumoniae* are bacterial co-pathogens usually detected (Lansbury et al., 2020). *Streptococcus*, *Klebsiella* and *Staphylococcu*s are generally involved in pulmonary cavitation (Aggarwal et al., 2021). Diffuse alveolar damage is proved to be the primary histopathological manifestation of lung cavities, and pulmonary fibrosis can also be noticed when cavities appear in the late phase of COVID-19 (Aggarwal et al., 2021). Lung cavities can be present in patients with both severe and mild COVID-19 (Chen et al., 2020a; Selvaraj and Dapaah-Afriyie, 2020; Afrazi et al., 2021; Aggarwal et al., 2021; Chen et al., 2021; Jafari et al., 2021; Zoumot et al., 2021). During the convalescent period of COVID-19, SARS-CoV-2 may colonize in alveolar necrotic tissue and fibrotic lung tissue by hiding inside biofilms, resulting in sustained injury and necrosis, and eventually the formation of lung cavities. SARS-Cov-2 may be hidden in biofilms for a long time, and possible latent sites include nasal sinuses, oral cavity, bronchoalveolar necrotic tissue, fibrotic lung tissue, etc.

## Pathogens’ abundance increases in the nose of COVID-19 patients

Rhoades et al. ‘s study found that the nasal microbiome of COVID-19 patients had an increase in bacterial pathogens, such as *Pseudomonas aeruginosa*, *Acinetobacter*, *Roche*, etc., and have positive correlation with SARS-CoV-2 RNA load (Rhoades et al., 2021). The presence of biofilms in the sinuses of experimental animals infected with *Pseudomonas aeruginosa* was identified by scanning electron microscopy (Vlastarakos et al., 2007). A systematic review of COVID-19 and *Mucor* co-infection shows that the most common co-infection spots are the nasal cavity, sinuses, and orbit, and can further co-infect with *Aspergillus* (SeyedAlinaghi et al., 2022). In this review, 45 (33.6%) out of 134 co-infected patients died, and related co-infection risk factors include diabetes, glucocorticoids therapy, immunomodulatory drugs use, immunocompromised status, hypertension, hematological malignancies, etc. Sufficient evidences have been documented to prove the existence of biofilm in the sinus mucosa of patients with chronic sinusitis (Bendouah et al., 2006; Palmer, 2006; Hunsaker and Leid, 2008; Bezerra et al., 2009; Kłodzińska et al., 2016; Michalik et al., 2018). There are more opportunistic bacteria and less symbiotic microorganisms in the nasal sinus of chronic sinusitis patients (Taylor et al., 2021). Biofilms can also be observed in healthy sinus mucosa (Vlastarakos et al., 2007; Mladina et al., 2010). We speculate that biofilms in nasal cavity and sinuses might harbor SARS-CoV-2, especially in patients with a history of chronic inflammation at these sites (**Figure 1**).

## SARS-CoV-2 are detected in dental biofilms

The periodontal pocket provides a unique subgingival environment and is an ideal location for pathogenic microorganisms to gather and produce biofilm. The dental root wall permits the formation of subgingival biofilms (Colombo and Tanner, 2019; Badran et al., 2020). SARS-CoV-2 has a certain resistance to the external environment, permitting its attachment to dental biofilms before being eliminated, which depends on the organic polymers such as polysaccharides, glycoproteins and lipids inside biofilms (Rabin et al., 2015; Scheller et al., 2020; Loveday et al., 2021; Samrot et al., 2021). Gomes’s team has confirmed the presence of SARS-CoV-2 in oral biofilm samples (Gomes et al., 2021). In this study, a total of 70 participants who had positive results for SARS-CoV-2 RNA from nasal/oropharyngeal swab samples using real-time quantitative polymerase chain reaction analysis were included. The result revealed that SARS-CoV-2 RNA were positive in 13 biofilm samples. Their another study showed that SARS-CoV-2 RNA was detected in oral biofilms in 26 out of 52 COVID-19 patients from the ICU, and it’s turn out that 96.2% positive samples were from subgingival specimens (Gomes et al., 2022). Furthermore, there are also other kinds of viral components within dental plaque (Das et al., 2012; Edlund et al., 2015), such as bacteriophage, herpes simplex virus (HSV) and Epstein-Barr virus (EBV) etc. Viruses in oral biofilms can also migrate to other spots through the bloodstream (Badran et al., 2020). SARS-CoV-2 may obtain strong resistance of viral drugs and human immune system and remain latent in the body for a long time by co-existing with other pathogenic microorganisms in subgingival biofilms. At the stage of biofilm detachment, due to anaerobic environment, nutrition deficiency and temperature altering, the secretion of dispersin B begin to increase (Samrot et al., 2021). Dispersin B is an enzyme which exists in the microbial extracellular matrix with the function of degrading polysaccharides in extracellular polymeric substances, permitting the detachment of adherent microorganisms (Samrot et al., 2021). We conjecture that SARS-CoV-2 can disengage from the biofilm along with bacteria, enter the oral cavity, mix with saliva, thus can be detected, and can also pass through the respiratory tract, reach the lung then cause cavitary lung lesions, and even spread to other parts through bloodstream, especially in immunocompromised patients (**Figure 1**).

## Pulmonary bacterial and fungal infections coexist with COVID-19

A great number of research studies have indicated that lung microbial communities include bacteria and other non-bacterial organisms, comprising fungi and viruses. SARS-CoV-2 may diffuse in the lung as aerosols, then colonize though pulmonary mucus (Nguyen et al., 2015; Chen et al., 2022). Gherlan et al. reported a case that the COVID-19 patient got the entire right lung removed, the direct microscopic detection confirmed *A. baumannii*, *Mucorales*, *Candida species* (Gherlan et al., 2022). Maccio et al. noticed accompanying bacterial pneumonia (23/35,66%) and diffuse alveolar damage (24/35,69%) in the autopsies of 35 COVID-19 patients, and lung aspergillosis was an associative double infection (Maccio et al., 2022). Edler et al. also found that some COVID-19 patients have bacterial superinfected bronchopneumonia (Edler et al., 2020). Chen et al. found *Veillonella* and *Capnocytophaga* in the bronchoalveolar lavage fluid (BALF) of COVID-19 patients (Chen et al., 2020b). Hedberg et al. discovered the presence of bacterial co-infection through the culture of samples from lower respiratory tract (Hedberg et al., 2022). In addition, some reports spot that COVID-19 can co-exist with tuberculosis or latent tuberculosis infection (Ata et al., 2020; Luke et al., 2022). Wang et al. collected BALF and sputum samples from COVID-19 patients and cultured *Aspergillus* (Wang et al., 2020a). Song et al.’ retrospective analysis indicates the presence of fungal and SARS-CoV-2 co-infections, particularly in immunocompromised and severe COVID-19 patients (Song et al., 2020). COVID-19 patients co-infected with *Candida, Mucor and Cryptococcus* have been reported as well (Zhu et al., 2020). Shen et al. found that there are similarities between the microorganisms of patients with community-acquired pneumonia (CAP) and COVID-19 patients, both contain an increasing number of symbiotic bacteria or are occupied by pathogens in oral and upper respiratory tract (Shen et al., 2020). Ren et al. detected *Pseudomonas*, *Streptococcus* and *Acinetobacter Baumannii* in the BALF of 5 hospitalized COVID-19 patients (Ren et al., 2020). Divisi et al. also determined the existence of *Pseudomonas aeruginosa* in COVID-19 patients’ pleural empyema (Divisi et al., 2021). SARS-CoV-2 can lead to a relatively anoxic environment in the lung, which is conducive to the presence and growth of oral anaerobe and facultative anaerobe (Bao et al., 2020). Hazardous factors, for instance, ventilation treatment, cough, increased inhalation and inferior oral hygiene offer opportunity for oral microbiome to migrate into lower respiratory tract (Bao et al., 2020).

## Discussion and conclusion

Characteristics of cases that SARS-CoV-2 co-infects with other pathogens include venerable age, glucocorticoid therapy, immunocompromised state, long-term antibiotic use history, diabetes, cardiovascular comorbidities, intensive care unit attending, ventilation treatment, prolonged hospitalization time, and exacerbation of symptoms (Bao et al., 2020; Blasco et al., 2020; Contou et al., 2020; Wang et al., 2020a; Alhumaid et al., 2021; He et al., 2021; Yasmin et al., 2021; Gomes et al., 2022; Hedberg et al., 2022; Ortega-Pena et al., 2022; Rouze et al., 2022; SeyedAlinaghi et al., 2022; Shetty et al., 2022). Bacterial pathogens generally isolated cover *M. pneumoniae*, *P. aeruginosa*, *H. influenzae*, *K. pneumoniae* and *S. pneumoniae* (Lansbury et al., 2020). *Streptococcus*, *Klebsiella* and *Staphylococcu*s are generally involved in pulmonary cavitation (Aggarwal et al., 2021). Langford et al. found bacterial co-infection and secondary bacterial infection account for 3.5% and 14.3% of COVID-19 patients respectively. Bacterial infection was found in 6.9% of COVID-19 patients overall, which was more frequent in seriously ill patients (Langford et al., 2020). A multi-center study showed that about 9.5% of the COVID-19 patients included in this study had clinically diagnosed bacterial co-infection, with an increased risk of bacterial co-infection in patients with advanced age or cardiovascular comorbidities (He et al., 2021). The total proportion of mortality in SARS-CoV-2 and fungal co-infection patients was 0.17, and there is an obvious distinction between the proportion of mixed hospitalized population and ICU patients (0.06 vs 0.36) (Peng et al., 2021). Contou et al. reported that 28% of severe COVID-19 patients had bacterial co-infection in their course of ICU hospitalization, related bacteria include *Enterobacteriaceae, Staphylococcus aureus*, *Streptococcus pneumoniae* and *Haemophilus influenzae* (Contou et al., 2020). The total mortality of COVID-19 patients with other pathogens co-infection increased significantly (Contou et al., 2020; Mirzaei et al., 2020; Alhumaid et al., 2021; He et al., 2021; Peng et al., 2021; Maccio et al., 2022; Rouze et al., 2022; SeyedAlinaghi et al., 2022). Many other studies have documented antibiotic resistance in COVID-19 patients with bacterial infections (He et al., 2021). The formation of biofilms can significantly enhance the resistance of microorganisms to antibiotics and the host immune attack, resulting in persistent and chronic infections, which is connected with high morbidity and mortality of diseases (Samrot et al., 2021). Some researchers recommend that patients be regularly screened for bacterial and fungal co-infections following a definite diagnose of SARS-CoV-2 infection (Zhu et al., 2020; White et al., 2021).

There are increasing cases of COVID-19 patients with cavitary lung lesions, re-positive or long-term positive nucleic acid tests (Chen et al., 2020a; He et al., 2020; Qiao et al., 2020; Selvaraj and Dapaah-Afriyie, 2020; Lu et al., 2020a; Lu et al., 2020a; Wang et al., 2020b; Aggarwal et al., 2021; Afrazi et al., 2021; Chen et al., 2021; Egoryan et al., 2021; Jafari et al., 2021; Liang et al., 2021; Ozgur and Dogan, 2021; Wu et al., 2021; Zhu et al., 2021; Zoumot et al., 2021; He et al., 2022; Maccio et al., 2022). Lung cavities appear at a long-time interval from initial novel coronavirus infection, generally during the absorption phase of the disease, may have severer symptoms after initial recovery and also an increasing mortality rate (Aggarwal et al., 2021; Chen et al., 2021; Egoryan et al., 2021; He et al., 2022; Zoumot et al., 2021). Maccio et al. performed autopsies on 35 COVID-19 patients and found that patients could still show diffuse alveolar damage 2 months after the initial diagnosis of COVID-19 (Maccio et al., 2022). Diffuse alveolar damage has a significant correlation with the persistence of SARS-CoV-2 RNA in the lung (Maccio et al., 2022). The main histopathological manifestations of lung cavities were also diffuse alveolar damage (Aggarwal et al., 2021). SARS-CoV-2 can multiply in alveolar epithelial cells and bronchiole mucosa, resulting in diffuse alveolar damage, alveolar cell hyperplasia, fibroblast hyperplasia and pulmonary fibrosis (Chen et al., 2021; Huang and Tang, 2021). These evidences suggest that SARS-CoV-2 may stay persistent and latent in bronchoalveolar necrotic tissue or fibrotic lung tissue. We hypothesize that occult SARS-CoV-2 may hide in the biofilm to evade the attack of antiviral drugs and the host immune system attack, and remain latent in the body, then, lead to cavitary lung lesions and re-positive nucleic acid tests through replication for a period of time (**Figure 1**). Persistent and repeated positive nucleic acid tests suggest that SARS-CoV-2 may retain for a long time and cause chronic lung damage, possibly associated with more serious pulmonary complications. The limitations about this hypothesis are that: the chance of contamination of upper respiratory population do exist during the bronchoalveolar lavage, although much measures have been adapted to minimize the possibility, such as the standard sterile procedure, the use of sterile reagent and the disinfectant immersion of the bronchoscope before use, we still cannot exclude the contamination completely (Pang et al., 1989). Besides, diffuse alveolar damage is not only the underlying pathophysiology of cystic or cavitary lung lesions, but the underlying pathophysiology of acute respiratory distress syndrome (ARDS) which represents a typical condition for severe COVID-19 patients. This kind of patients usually need mechanical ventilation which itself can induce barotrauma and ventilator associated lung injury (VILI). Thus, being a potential factor in the formation of cystic or cavitary pulmonary lesion (Cardinal-Fernandez et al., 2017). But the existence of some mild COVID-19 cases with cystic or cavitary lung lesions relatively increases the possibility of SARS-CoV-2 directed lung lesions for the low chance of ARDS in mild cases (Chen et al., 2021; He et al., 2022).

Some COVID-19 patients had an elevated level of inflammatory cytokines and biomarkers associated with bacterial co-infection (Mirzaei et al., 2020). Joseph et al. proposed that bioluminescent imaging technique may be considered as a promising method to confirm the relevant biofilm load of COVID-19 patients, providing new strategy for clinical diagnosis (Joseph and Steier, 2022). The hypothesis that SARS-CoV-2 may co-exist in biofilms with other microorganisms depends on available evidences and clinical observation. If this hypothesis is confirmed, future researches are supposed to focus on finding suitable biomarkers or applying new techniques to help identify SARS-CoV-2 hiding in biofilms and using antibiofilm agents (such as bioactive compounds) in combination concerning COVID-19 treatment.

## Author contributions

DH and CF wrote the initial manuscript. DH drew the figure. MN and XH revised the manuscript. YC and SS coordinated, supervised, and critically reviewed the manuscript for important intellectual content. All authors listed have made a substantial, direct, and intellectual contribution to the work and approved it for publication.

## Funding

National Natural Science Foundation of China (No: 82272980, 82060426); Yunnan Health Training Project of High-Level Talents (H-2018025).

## Conflict of interest

The authors declare that the research was conducted in the absence of any commercial or financial relationships that could be construed as a potential conflict of interest.

## Publisher’s note

All claims expressed in this article are solely those of the authors and do not necessarily represent those of their affiliated organizations, or those of the publisher, the editors and the reviewers. Any product that may be evaluated in this article, or claim that may be made by its manufacturer, is not guaranteed or endorsed by the publisher.
